# The BG Study Part 1 (Bergisch Gladbach): Development of a Prototype Coronary Artery Disease Risk Score Incorporating Peripheral Vascular Parameters—Preliminary Insights for Future CAD Risk Prediction Models in Vascular Patients

**DOI:** 10.3390/jcm14041297

**Published:** 2025-02-15

**Authors:** Tuna Aras, Mahmoud Tayeh, Adel Aswad, Mohamed Sharkawy, Zaki Almuzakki, Bernhard Dorweiler, Payman Majd

**Affiliations:** 1 Department of Vascular Surgery, EVK Bergisch Gladbach, Ferrenbergstraße 24, 51465 Bergisch Gladbach, Germany; m.tayeh@evk.de (M.T.); p.majd@evk.de (P.M.); 2 Al-Qassimi Teaching Hospital and Cardiac Centre, University of Sharjah, Sharjah P.O. Box 3500, United Arab Emirates; vascular1984@gmail.com (A.A.); zaki.almuzakki@gmail.com (Z.A.); 3 Department of Vascular Surgery, Faculty of Medicine, Cairo University Hospital, Cairo 4240310, Egypt; sharkawy54@me.com; 4 Department of Vascular and Endovascular Surgery, Faculty of Medicine, University Hospital of Cologne, University of Cologne, 50937 Cologne, Germany; bernhard.dorweiler@uk-koeln.de

**Keywords:** coronary artery disease, peripheral artery disease, ankle–brachial index, carotid disease, risk score

## Abstract

**Background:** Peripheral vascular parameters may provide valuable insights into coronary artery disease (CAD) risk stratification. This study aimed to develop a CAD risk score by integrating carotid duplex velocities, ankle–brachial index (ABI), and clinical history into a point-based model. **Methods:** We analyzed data from 902 cardiology patients, of whom 592 (65.6%) had confirmed CAD based on coronary angiography. Peripheral vascular assessments included carotid duplex ultrasonography and ABI measurements. Predictors were identified through multivariate logistic regression, addressing multicollinearity and interaction effects. A point-based scoring system was developed using statistically significant variables and evaluated via receiver operating characteristic (ROC) analysis. **Results:** Key predictors included external carotid artery velocities, ABI, carotid stenosis, chronic kidney disease (CKD) stage, smoking history, diabetes, hypertension, and age. The scoring system demonstrated moderate discriminative ability (AUC: 0.683) and high sensitivity (97%) for detecting CAD-positive cases but lower specificity (11%) for CAD-negative cases. Patients were stratified into risk categories, with an optimal threshold of ≥7 points maximizing the F1 score. **Conclusions:** This novel scoring system highlights the clinical relevance of integrating peripheral vascular assessments into CAD risk models. While its high sensitivity ensures robust detection of CAD-positive patients, future multicenter studies are needed to improve specificity and validate its broader clinical utility.

## 1. Introduction

Coronary artery disease (CAD) and peripheral arterial disease (PAD) are often treated as distinct conditions despite their shared pathophysiological origins as manifestations of systemic atherosclerosis. Traditional CAD risk models, including the Framingham Risk Score [1] and CAD Consortium [2] algorithms, primarily rely on clinical and biochemical markers while neglecting peripheral vascular assessments such as carotid velocities and the ankle–brachial index (ABI). This compartmentalized approach may overlook systemic indicators of vascular pathology, limiting diagnostic accuracy and risk stratification.

Emerging evidence suggests that integrating peripheral vascular findings could enhance CAD prediction, particularly in populations with overlapping vascular conditions. Carotid ultrasound, for example, provides hemodynamic insights often missed by ABI, which may remain normal despite the underlying pathology. Yet, peripheral markers remain underutilized in clinical practice and risk prediction models.

This study addresses these gaps by developing and validating a novel CAD risk scoring system—the BG scoring system—that incorporates peripheral vascular parameters alongside traditional clinical predictors. We hypothesized that integrating these data would improve CAD detection, particularly in high-risk populations, and provide a more comprehensive framework for early intervention.

## 2. Methods

This study prospectively analyzed data from patients admitted to the cardiology department for the evaluation of coronary artery disease (CAD) between 2021 and 2023. All patients underwent coronary angiography due to their presenting symptoms, and the diagnosis or exclusion of CAD was determined by cardiologists. Peripheral vascular assessments, including carotid duplex ultrasonography and ankle–brachial index (ABI) measurements, were performed using standardized protocols. Patients with incomplete medical records were excluded from the analysis. Ethical approval was obtained from the Ethics Committee of the Ärztekammer Nordrhein-Westfalen (Registration No. 2020300), and informed consent was secured from all participants. Ultrasonographic assessments were conducted using a single ultrasound device (Philips Affiniti 30, Philips Healthcare, Amsterdam, Netherlands) to ensure consistency in imaging quality. Statistical analyses were performed using IBM SPSS Statistics version 28.0 (IBM Corp., Armonk, NY, USA) and Python version 3.9 (Python Software Foundation, Wilmington, DE, USA; https://www.python.org, accessed on 15 December 2024), with key Python libraries including NumPy (v1.21.0) (NumPy Developers), SciPy (v1.7.0) (SciPy Developers), pandas (v1.3.0) (pandas Development Team), and statsmodels (v0.13.0) (statsmodels Developers). The study hypothesized that peripheral vascular parameters could aid in CAD detection. Variables considered for the predictive scoring system were selected based on statistical analyses and included hypertension, antihypertensive medication use, internal carotid artery stenosis, chronic kidney disease (CKD) at stage 3b or higher, smoking history, angina, peripheral artery disease (PAD), diabetes, age, and external carotid artery velocities. Internal carotid artery stenosis was categorized into mild (<50%), moderate (50–69%), and severe (≥70%), while external carotid artery velocities were analyzed as continuous variables to capture hemodynamic changes. To ensure model stability, multicollinearity was assessed using Variance Inflation Factor (VIF) analysis, with variables exhibiting VIF > 10 excluded. Stepwise regression (backward selection, *p* < 0.05) and correlation matrices were employed to refine predictor selection, ensuring a balance between statistical significance and clinical relevance. Marginally significant predictors were retained if they demonstrated consistent trends across multiple analyses or provided meaningful clinical insights, such as smoking history. Interaction effects between variables were also evaluated to improve model performance. Pearson’s correlation coefficient was used for continuous variables, while chi-square tests assessed categorical associations. Logistic regression models were applied to identify key predictors of CAD, calculating odds ratios (ORs) with 95% confidence intervals (CIs). Multivariate models were adjusted for potential confounders, including age, sex, diabetes, and hypertension. To evaluate the discriminatory power of the scoring system, receiver operating characteristic (ROC) curve analysis was conducted, with the area under the curve (AUC) used to quantify model performance. AUC values were classified as poor (0.50–0.69), moderate (0.70–0.79), good (0.80–0.89), and excellent (≥0.90). Additionally, precision-recall curve analysis was performed to optimize threshold selection by maximizing the F1 score, ensuring robust classification performance.

## 3. Results

We analyzed data from 902 patients admitted for coronary artery disease (CAD) evaluation. Among them, 592 (65.6%) were diagnosed with CAD—190 (21.0%) had single-vessel disease, 189 (20.9%) had two-vessel disease, and 213 (23.6%) had three-vessel disease.

A total of 311 patients (34.5%) had a prior CAD diagnosis and were readmitted for symptom progression, while 310 (34.4%) were ruled out for CAD. Previous revascularization for CAD was recorded in 278 patients (30.8%). Table 1 summarizes the key results.

**Figure 1:**
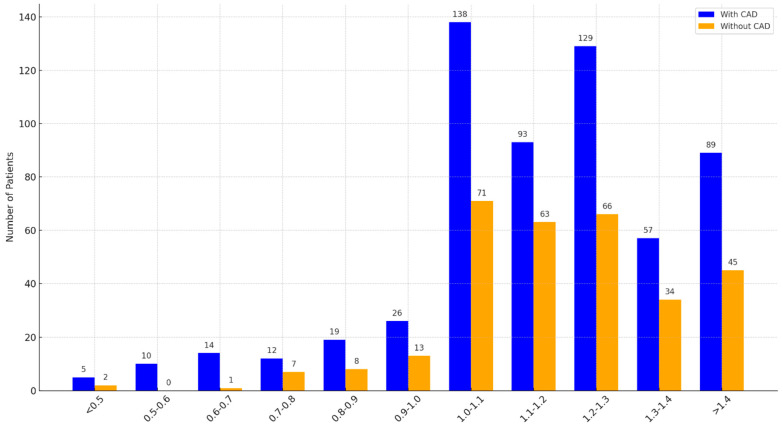
The bar chart shows the prevalence of coronary artery disease (CAD) in patients categorized by ankle–brachial index (ABI) ranges. Blue bars represent patients with CAD, and orange bars represent patients without CAD. Each bar is annotated with the exact number of patients within the respective ABI range.

Ankle–brachial index (ABI) analysis showed the strongest CAD associations in ranges of 0.5–0.6 (*p* = 0.049, OR = 2.23, 95% CI: 1.00–4.98) and 0.6–0.7 (*p* = 0.045, OR = 2.07, 95% CI: 1.01–4.23), although smaller sample sizes in lower ABI ranges may have affected reliability (Figure 1).

We analyzed the peripheral vascular status of the 311 patients with a prior CAD diagnosis. A total of 93 patients (29.9%) had previously diagnosed PAD, while 13 patients (4.18%) had no prior PAD but were newly diagnosed with either PAD, low ABI (<0.9), or high-grade carotid stenosis in this study. These findings emphasize the importance of peripheral vascular parameters in CAD patients and their potential impact on overall cardiovascular risk assessment and the temporal progression of atherosclerotic vascular disease.

**Figure 2:**
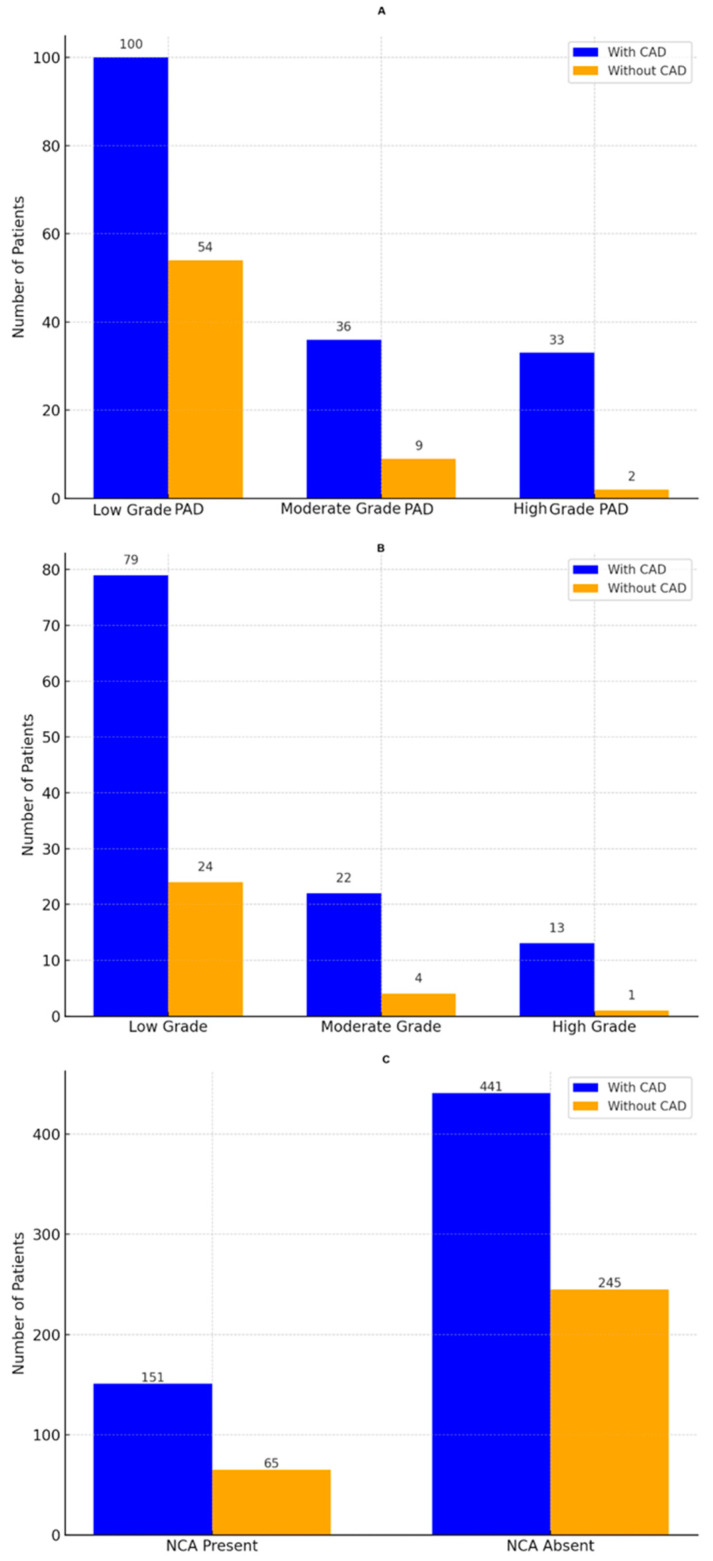
The figure presents the prevalence and associations of coronary artery disease (CAD) across different vascular conditions. Panel (**A**) illustrates the distribution of CAD among patients with low-, moderate-, and high-grade peripheral arterial disease (PAD). Blue bars represent patients with CAD, while orange bars represent those without CAD, with patient counts displayed above each bar. Panel (**B**) depicts CAD prevalence across internal carotid artery stenosis grades (low, moderate, and high). Blue bars correspond to CAD-positive patients, while orange bars correspond to CAD-negative patients, with the total number of patients annotated for clarity. Panel (**C**) highlights the association of non-compressible arteries (NCAs) with CAD, showing the number of patients with and without CAD in groups with NCAs present and NCAs absent. Blue bars represent CAD-positive patients, while orange bars indicate CAD-negative patients, with exact patient counts labeled above each bar.

Among 902 patients, 343 (38.0%) were lifelong non-smokers, 359 (39.8%) were former smokers, and 200 (22.2%) were current smokers. PAD severity was categorized into three grades: mild, moderate, and severe. Mild PAD corresponded to claudication occurring after walking more than 200 m, moderate PAD was defined by claudication occurring within 200 m, and severe PAD included cases with rest pain, tissue loss, or claudication after only a few meters (Figure 2A).

Non-compressible arteries, present in 216 patients (23.9%), were not linked to CAD (*p* = 0.374) but were associated with PAD (*p* = 0.033, OR = 1.94, 95% CI: 1.06–3.55; Figure 2C).

Carotid stenosis, assessed using NASCET criteria, was significantly associated with CAD (*p* = 0.00016, OR = 2.95, 95% CI: 1.73–5.01), with higher CAD prevalence in patients with higher-grade stenosis (Figure 2B).

**Figure 3:**
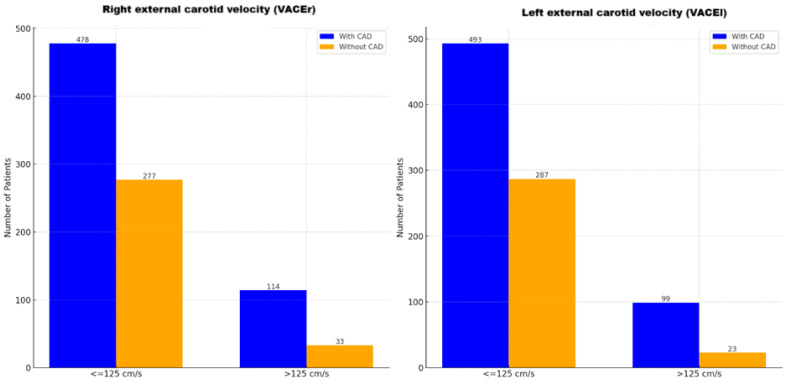
Association between left and right external carotid artery velocities (VACEl, VACEr) and coronary artery disease (CAD) (≤125 cm/s vs. >125 cm/s). The bar charts display the distribution of CAD among patients based on right external carotid velocity (VACEr) and left external carotid velocity (VACEl). Blue bars represent patients with CAD, while orange bars represent patients without CAD. Carotid velocities are categorized for analysis into two groups: ≤125 cm/s and >125 cm/s.

External carotid artery velocities were highly associated with CAD (left: *p* < 0.00001, OR = 2.83, 95% CI: 1.72–4.63; right: *p* < 0.00003, OR = 2.71, 95% CI: 1.63–4.49; Figure 3). Lower-grade stenosis in the internal carotid arteries also correlated with CAD (left: *p* = 0.011, OR = 1.64, 95% CI: 1.12–2.39; right: *p* = 0.030, OR = 1.58, 95% CI: 1.06–2.35; Figure 2B). However, common carotid artery velocities showed no significant association (left: *p* = 0.763; right: *p* = 0.711).

**Figure 4:**
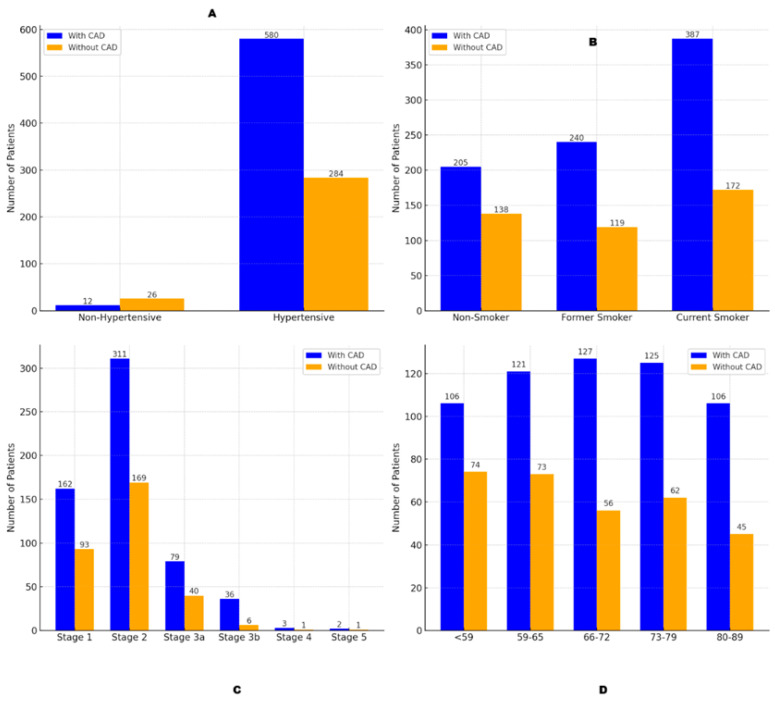
This figure shows the relationship between CAD prevalence and key demographic and lifestyle risk factors: hypertension, smoking history, CKD stages, and age. Panel (**A**): CAD prevalence in hypertensive versus non-hypertensive patients, with a significantly higher prevalence in hypertensive individuals. Panel (**B**): CAD prevalence across smoking history categories, highlighting the highest prevalence in current smokers. Panel (**C**): CAD prevalence across CKD stages, with advanced CKD stages showing a marked increase in CAD risk. Panel (**D**): CAD prevalence by age group, demonstrating an age-related increase in CAD prevalence, with the highest rates in the oldest age group.

Hypertension was significantly associated with CAD, affecting 67.1% of hypertensive patients (580 of 864) compared to 31.6% of non-hypertensive patients (12 of 38; *p* < 0.001, OR = 4.05, 95% CI: 2.15–7.64; Figure 4A). A linear correlation (r = 0.96) between CAD prevalence and the number of antihypertensive medications further highlighted this relationship.

Smoking also increased CAD risk, particularly among current smokers (*p* = 0.008, OR = 1.91, 95% CI: 1.18–3.10), with cumulative exposure (pack years) showing a positive correlation (*p* = 0.003, OR = 1.04, 95% CI: 1.01–1.07; Figure 4B).

Chronic kidney disease (CKD) stages also correlated with CAD, with significant increases beginning at Stage 3b (*p* = 0.004, OR = 3.94, 95% CI: 1.51–10.27), compared to Stage 2 (*p* = 0.018, OR = 3.56, 95% CI: 1.24–10.21) and Stage 3a (Figure 4C). Severe stages (4 and 5) lacked statistical significance due to small sample sizes.

Age was a key predictor, with CAD prevalence rising across groups: 50.0% under 59 years, 59.2% at 59–65 years, 68.4% at 66–72 years, 67.2% at 73–79 years, and 73.5% at 80–89 years (*p* = 0.027; Figure 4D). Age also increased the likelihood of CAD and PAD co-occurrence (*p* < 0.0001), reflecting a progressive cardiovascular risk with advancing age.

To develop the coronary artery disease (CAD) risk scoring system, we selected statistically significant predictors identified through analysis. Key variables included the number of antihypertensive medications, hypertension, carotid stenosis, CKD Stage 3b or higher, smoking history, angina pectoris, PAD in anamnesis, diabetes, age, and carotid artery stenosis. Internal carotid stenosis was categorized into low, moderate, and high grades based on clinical guidelines, while external carotid velocities were treated as continuous variables to capture subtle hemodynamic differences linked to CAD risk. Table 2 presents the point-based scoring system.

Points were assigned to each variable, and patients were stratified into four risk categories: 0–3 points (low risk), 4–6 points (moderate risk), 7–10 points (high risk), and 11+ points (very high risk).

ROC curve analysis demonstrated moderate discriminative ability with an area under the curve (AUC) of 0.683 (Supplementary Figure S1). Precision-recall curve analysis identified seven points as the optimal scoring threshold, maximizing the F1 score. The system showed high sensitivity for CAD-positive patients (97%) but lower specificity for CAD-negative cases (11%), resulting in an overall accuracy of 68%.

## 4. Discussion

This study examines the challenges and potential benefits of integrating peripheral vascular parameters into CAD evaluation. Such integration could optimize the use of non-invasive and invasive diagnostics in high-risk populations, enhancing resource efficiency and diagnostic accuracy.

Our discussion will primarily focus on findings related to the ankle–brachial index (ABI), peripheral arterial disease (PAD), and carotid parameters, as the relationship between atherosclerotic cardiovascular disease and factors such as hypertension, chronic kidney disease (CKD), smoking, age, and diabetes—elements already incorporated into our scoring system—is well established [3,4,5,6,7].

Our patient cohort consists of individuals admitted to the cardiology department for the assessment of CAD, representing a relatively younger population compared to patients in a vascular surgery department. A significant challenge in understanding the connection between coronary artery disease (CAD) and peripheral artery disease (PAD) lies in their differing timelines of progression. CAD typically develops earlier in the disease trajectory, whereas PAD often begins as silent atherosclerosis, manifesting clinically at older ages, with symptoms such as claudication or chronic limb-threatening ischemia (CLTI) [8,9]. This disparity arises from physiological differences between the heart muscle and the peripheral skeletal muscle. The heart’s constant and high demand for oxygen and nutrients makes it highly susceptible to ischemia, leading to early clinical manifestations such as angina or myocardial infarction [10].

The temporal progression of atherosclerotic cardiovascular disease is further influenced by additional factors such as genetic [11,12], epigenetic [13], environmental [14,15], and ethnic variables [16,17], which add significant complexity to understanding its progression and interconnectedness (Supplementary Figure S2). These factors shape the onset, progression, and clinical manifestations of atherosclerosis, varying widely across populations and individuals. Epigenetic modifications, driven by environmental exposures and lifestyle choices, can accelerate or delay disease progression, while ethnic variations contribute to differences in vascular biology, risk factor prevalence, and disease expression.

In CAD, a critical narrowing of the coronary arteries may lead to stable angina, which occurs when myocardial oxygen demand exceeds supply but remains chronic and predictable in the presence of significant stenosis. However, acute coronary syndromes (ACSs) occur when the rupture of atherosclerotic plaque triggers thrombosis, leading to plaque rupture (NSTEMI) or total occlusion (STEMI). These acute events often present with severe symptoms that prompt immediate medical evaluation, leading to rapid disease detection.

Conversely, PAD follows a more gradual trajectory, characterized by progressive arterial narrowing and a slow reduction in perfusion pressure to the affected extremities. This results in intermittent claudication, a symptom that patients often tolerate for long periods, attributing it to orthopedic conditions or age-related decline. Many patients continue ambulating, which promotes collateral vessel formation and temporarily alleviates symptoms, creating a false impression of healing. However, as the disease progresses, pain worsens and eventually becomes intolerable. Additionally, the occurrence of appositional thrombosis in narrowed arteries can lead to an acute worsening of symptoms or critical ischemia of the extremity, prompting late-stage diagnosis. This explains why the literature consistently reports a very high prevalence of CAD in PAD patients, while PAD prevalence in CAD cohorts tends to be lower [7,18,19]. This observation was also evident in our study, where the cohort predominantly exhibited normal or only slightly abnormal ABI values. This highlights the necessity for a deeper exploration of these interconnections, with particular attention to the varying timelines of hemodynamically significant atherosclerosis development across different arterial beds, to facilitate the translation of findings into enhanced clinical care.

An important consideration in interpreting our findings is the presence of angina in 55.5% of patients without angiographically significant CAD, which warrants further discussion. One potential contributing factor is the impact of the COVID-19 pandemic, as data collection occurred between 2021 and 2023. COVID-19 has been associated with persistent cardiovascular symptoms, including microvascular dysfunction, endothelial inflammation, and autonomic dysregulation, which may contribute to angina, ischemic ECG changes, and elevated cardiac biomarkers in the absence of obstructive CAD.

Beyond COVID-19, several other conditions may explain ischemic symptoms and abnormal cardiac markers in CAD-negative patients, such as microvascular angina (INOCA) and vasospastic angina (Prinzmetal’s angina). Additionally, myocarditis, pericarditis, chronic kidney disease (CKD), and hypertensive heart disease can lead to ECG abnormalities and biomarker elevations due to myocardial inflammation or increased cardiac stress. These factors probably contribute to the observed 55.5% of patients without angiographically significant CAD who experienced angina.

The ankle–brachial index (ABI) demonstrated a significant association with coronary artery disease (CAD) in our primary analysis, particularly within the ABI ranges of 0.5–0.6 and 0.6–0.7. However, this predictive power diminished in the logistic regression analysis, probably due to the high specificity but relatively low sensitivity of ABI measurements for predicting vascular risk—a limitation well documented in the literature [20,21,22,23,24,25,26]. Moreover, the smaller sample sizes within the lower ABI categories in this study may have further reduced the statistical power of these associations.

The ranges of 0.5–0.6 and 0.6–0.7 demonstrated the strongest associations with CAD in our univariate analysis, supporting their inclusion as distinct categories. However, ABI values below 0.5, which often represent advanced PAD, were underrepresented in our cohort, potentially limiting the statistical power of these categories. Additionally, the inclusion of non-compressible arteries as a separate category addressed the unique hemodynamic profile of patients with advanced vascular calcification, a condition frequently associated with PAD but requiring distinct consideration due to its interference with the accuracy of ABI measurement.

Our findings emphasize the temporal and pathophysiological differences between coronary artery disease (CAD) and peripheral arterial disease (PAD). In summary, CAD tends to present earlier due to the heart’s high oxygen demand and susceptibility to ischemia, which is also a well-recognized symptom in the general population. In contrast, PAD often progresses silently, frequently masked or compensated by well-developed collateral circulation. This can lead to delayed recognition or misattribution of symptoms to other conditions, such as lumbar or spinal pathologies. Consequently, PAD typically manifests later, following extensive arterial calcification and diminished collateralization. This disparity in progression explains the lower prevalence of advanced PAD in our CAD cohort, where patients were younger, exhibited less severe PAD, and had higher ABI values. The relationship between CAD and PAD as manifestations of systemic atherosclerosis cannot be overlooked. This highlights the need to incorporate newer and novel modalities for the detection of PAD and the assessment of its severity to better reveal the interconnectedness of CAD and PAD.

Incorporating diagnostic modalities—such as imaging markers (e.g., femoral artery plaques, pulse wave velocity, and pressure–volume recordings) and ultrasonographic markers (e.g., longitudinal plaque displacement)—could further enhance diagnostic sensitivity and provide a more comprehensive assessment. These advancements may facilitate the development of robust and adaptable risk prediction models suitable for diverse clinical settings.

Another important finding from the study is that patients with a history of PAD, prior PAD-specific therapy, or advanced symptoms (e.g., claudication or chronic limb-threatening ischemia) exhibited a significant association with CAD. This highlights the proportional relationship between the severity of PAD and the likelihood of CAD, a correlation also well documented in the literature [18,21,27].

Not only a low ABI but also a high ABI can serve as evidence of advanced atherosclerosis, particularly in cases of medial sclerosis [28]. Although we did not find a significant association between the non-compressibility of arteries and coronary artery disease (CAD), there was a notable association with peripheral artery disease (PAD).

ABI, as a pressure-based measurement, is further limited by its susceptibility to influence from collateral circulation. In cases of well-collateralized stenoses or occlusions, normal resting ABI values can mask underlying PAD, reducing the sensitivity of ABI in detecting atherosclerotic disease [29,30]. Despite these limitations, ABI remains a valuable parameter for assessing systemic atherosclerosis when integrated with other diagnostic modalities. For example, ultrasonographic assessment of femoral plaque burden [31] or brachial–ankle pulse wave velocity (baPWV) [32] and longitudinal plaque displacement [33] could complement ankle–brachial index (ABI) measurements to provide a more comprehensive evaluation of vascular disease.

Our findings demonstrated a strong association between carotid artery pathology and coronary artery disease (CAD), aligning with prior research. Carotid velocity thresholds were based on established Doppler ultrasound standards correlating velocity with stenosis severity. External carotid artery velocities, treated as continuous variables, effectively captured subtle hemodynamic changes associated with CAD risk. This approach improved statistical differentiation between CAD-positive and CAD-negative patients, minimizing the loss of predictive power that can result from discrete categorization. For internal carotid artery stenosis, thresholds were categorized into low, moderate, and high grades according to NASCET [34] criteria, aligning with clinical guidelines.

Research conducted both in Japan and in western countries has demonstrated a high prevalence of carotid stenosis among patients with CAD, highlighting the importance of screening for carotid artery stenosis in this population [35,36]. Additionally, the presence and extent of carotid plaques have been linked to a long-term risk of coronary heart disease and the development of coronary artery calcification (CAC) among asymptomatic middle-aged individuals with an initial CAC score of 0 [37]. In patients undergoing evaluation for chest pain, carotid disease has been strongly associated with severe CAD [38]. Moreover, the burden of carotid plaque appears to have a stronger correlation with CAD than with arterial stiffness, as indicated by another study [39]. Interestingly, research involving 153 Chinese patients with a history of diabetes, hypertension, transient ischemic attack (TIA), stroke, or peripheral vascular disease (PVD) revealed that such individuals are more likely to exhibit internal carotid stenosis alongside concomitant vertebral and external carotid artery stenosis [40]. The PRECORIS study further underscored the relevance of cervicocephalic artery stenosis, showing that stenosis ≥50% in these arteries strongly correlates with asymptomatic CAD of similar severity [41]. Additionally, a retrospective cohort study of 38,201 patients who underwent carotid duplex ultrasound at a medical center in Taiwan identified carotid artery diameter reduction as an independent risk factor for both all-cause and cardiovascular mortality [42]. Lastly, a 2021 meta-analysis emphasized the shared pathophysiological mechanisms underlying atherosclerosis in the carotid and coronary systems. This underscores the value of carotid artery examination in patients suspected of having CAD, as it may provide critical diagnostic and prognostic insights [43].

An intriguing consideration in this context is the comparative utility of carotid ultrasound versus the ankle–brachial index (ABI) for vascular assessment. While ABI—a physiological pressure measurement—may appear normal in some cases, potentially masking peripheral arterial disease, carotid plaque imaging has consistently demonstrated predictive reliability. This finding supports its inclusion in our scoring system and underscores the need for developing additional novel assessment methods for the detection and severity evaluation of PAD. Integrating carotid plaque visualization into routine clinical practice could enhance the early identification of patients at high risk for CAD, particularly in populations with overlapping vascular conditions.

Direct imaging and hemodynamic evaluation of atherosclerotic plaques, using modalities such as carotid or femoral ultrasound, appear to provide more reliable diagnostic information than physiological tests like the ABI. Supporting this assertion, previous research demonstrated that femoral ultrasound achieved a sensitivity of 85% for detecting significant CAD, compared to just 25% for the ABI [31]. In a logistic regression analysis, the screening of atherosclerotic plaques yielded a statistically significant area under the curve (AUC) for CAD prediction at 0.812 (95% CI, *p* < 0.001), whereas neither ABI measurement nor carotid intima–media thickness (CIMT) achieved statistically significant performance in predicting CAD [44].

Moreover, three independent studies have highlighted that multiterritorial plaque quantification offers superior predictive value for CAD. This underscores the importance of utilizing comprehensive plaque imaging approaches to enhance the accuracy of CAD risk stratification and diagnosis [45,46,47].

In developing the coronary artery disease (CAD) scoring system, we prioritized sensitivity to minimize false negatives, particularly in high-risk populations such as vascular surgery candidates. This approach achieved a sensitivity of 97%, ensuring robust detection of CAD-positive cases. However, this emphasis on sensitivity came at the expense of specificity (11%) and led to a higher rate of false positives.

Receiver operating characteristic (ROC) analysis (AUC: 0.683) demonstrated moderate discriminative ability, consistent with the emphasis on sensitivity. Precision-recall analysis identified a seven-point threshold to maximize the F1 score, balancing sensitivity and specificity. While this ensures high recall for CAD-positive cases, future refinements are needed to enhance specificity without compromising sensitivity.

The BG scoring system uniquely integrates peripheral vascular parameters such as peripheral artery disease and carotid velocities—features absent in many existing models.

In contrast, systems like CAD-RADS rely primarily on coronary CT angiography. A comparison of variables across different scoring systems is summarized in Supplementary Table S1.

The BG scoring system demonstrates high sensitivity but lower specificity compared to the other systems. Models like CAD-RADS [48] achieve better discrimination (AUC ~0.86) due to their reliance on imaging data. In contrast, the Diamond–Forrester [2] score shows the weakest performance, with an AUC of ~0.58. The performance metrics of each scoring system are summarized in Supplementary Table S2 [1,49,50,51,52].

The BG study focused on a cardiology cohort with comprehensive vascular assessments, whereas the Diamond–Forrester and CAD Consortium scores were validated in outpatient populations with stable chest pain. The Duke Clinical Score [53] incorporated stress testing for hospital and outpatient settings, while the Framingham Risk Score addressed long-term cardiovascular risk in primary care. CAD-RADS, in contrast, is an imaging-based system optimized for coronary CT angiography.

Factors significant in univariate analyses often lose predictive strength in multivariate models due to multicollinearity, interaction effects, and small sample sizes. In this study, variables like ABI ranges and lower-grade carotid stenosis initially showed significance but lost predictive power in multivariate analysis, highlighting the complexity of systemic diseases like CAD and the need for rigorous methodology and an integrated clinical approach.

Predicting CAD across diverse populations requires tailored scoring systems, as performance may vary significantly in vascular surgery candidates, older patients, and different ethnic groups. These variations pose challenges for developing a universal model with both high sensitivity and specificity.

Future iterations should refine variable selection, incorporate biomarkers and advanced imaging parameters, and leverage machine learning to address multicollinearity and interaction effects. Multicenter validation in diverse cohorts will be essential to enhance generalizability and optimize the scoring system’s clinical utility.

## 5. Conclusions

The BG scoring system introduces an innovative approach to coronary artery disease (CAD) risk assessment by integrating peripheral vascular parameters, such as peripheral artery disease history, carotid velocities, and traditional clinical factors. Highlighting the systemic nature of atherosclerosis, it demonstrated high sensitivity (97%) in detecting CAD-positive patients.

Despite its strengths, the system’s low specificity (11%) raises concerns about false positives, potentially leading to unnecessary testing in CAD-negative patients. Moreover, its moderate discriminative ability (AUC: 0.683) highlights the need for further refinement to enhance predictive accuracy. Additionally, the single-center design limits the generalizability of our findings.

Nevertheless, the model shows promise, especially in low-resource settings where advanced diagnostics are limited. Future iterations could leverage machine learning (ML) and artificial intelligence (AI) to refine variable interactions, address multicollinearity, and optimize sensitivity-specificity balance. AI-driven approaches may also personalize risk assessment by dynamically adjusting thresholds based on patient profiles, enhancing accuracy and clinical relevance.

In conclusion, the BG scoring system provides a valuable framework for integrating peripheral vascular parameters into CAD risk assessment. While it excels in sensitivity and highlights systemic vascular disease, future refinements, validation in broader cohorts, and AI-based enhancements could strengthen its clinical utility and expand its applicability across diverse healthcare settings.

## Figures and Tables

**Table 1:** This table summarizes the baseline characteristics of the study population, stratified by the presence or absence of coronary artery disease (CAD). Continuous variables, such as age, are presented as mean ± standard deviation. Categorical variables, including smoking status, hypertension, diabetes mellitus, peripheral arterial disease (PAD), carotid stenosis, and angina pectoris, are expressed as absolute numbers with corresponding percentages. Percentages represent the proportion of patients within each subgroup (with CAD or without CAD).

Variable	With CAD (*n* = 592)	Without CAD (*n* = 310)
Age (years) (mean ± SD)	69.4 ± 10.7	66.6 ± 11.3
Male (%)	422 (71.3%)	198 (63.9%)
Female (%)	170 (28.7%)	112 (36.1%)
Smoking—Current (%)	387 (65.4%)	172 (55.5%)
Smoking—Former (%)	240 (40.5%)	119 (38.4%)
Smoking—Never (%)	205 (34.6%)	138 (44.5%)
Hypertension (%)	580 (98.0%)	284 (91.6%)
Non-Hypertension (%)	12 (2.0%)	26 (8.4%)
Diabetes Mellitus (%)	191 (32.3%)	68 (21.9%)
Peripheral Arterial Disease—Yes (%)	169 (28.5%)	65 (21.0%)
Peripheral Arterial Disease—No (%)	423 (71.5%)	245 (79.0%)
Carotid Stenosis—Yes (%)	133 (22.5%)	38 (12.3%)
Angina Pectoris—Yes (%)	389 (65.7%)	172 (55.5%)

**Table 2:** Point-based scoring system for predicting coronary artery disease (CAD). Abbreviations: CKD, chronic kidney disease; NASCET, North American Symptomatic Carotid Endarterectomy Trial; PAD, peripheral arterial disease; VACEl, velocity in the left external carotid artery; VACEr, velocity in the right external carotid artery.

Variable	Criteria	Points
Amount of Antihypertensive Medications	0, 1, 2, 3+ medications	0, 1, 2, 3
Hypertension	No, Yes	0, 1
Carotid Stenosis (according to NASCET)	≤50%, >50% stenosis	0, 2
CKD Stage	Stage ≤ 2, Stage 3, Stage ≥ 3b	0, 1, 2
Smoking History	Non-smoker, Smoker	0, 1
Angina Pectoris	No, Yes	0, 2
PAD History (Anamnesis)	No PAD, PAD Present (any severity)	0, 2
Diabetes	No, Yes	0, 2
Age	<50, 50–59, 60–69, 70+ years	0, 1, 2, 3
Carotid Velocity (VACEl)	≤125 cm/s, >125 cm/s	0, 1
Carotid Velocity (VACEr)	≤125 cm/s, >125 cm/s	0, 1

## Data Availability

The data is not available due to privacy and ethical restriction.

## Supplementary Materials

The following supporting information can be downloaded at: https://www.mdpi.com/article/10.3390/jcm14041297/s1, Figure S1: Receiver Operating Characteristic (ROC) curve evaluating the performance of the risk score in predicting CAD in our cohort; Figure S2: This figure illustrates the temporal progression and relationship between coronary artery disease (CAD) and peripheral artery disease (PAD), highlighting the factors influencing their development and progression; Table S1: Variables used in the BG scoring system and other coronary artery disease (CAD) risk scoring models; Table S2: Performance metrics of the BG scoring system compared to other coronary artery disease (CAD) risk scoring models.
