# Supplementary material for: The BG Study Part 1 (Bergisch Gladbach): Development of a Prototype Coronary Artery Disease Risk Score Incorporating Peripheral Vascular Parameters—Preliminary Insights for Future CAD Risk Prediction Models in Vascular Patients
Source: J Clin Med. 2025 Feb 15;14(4):1297. doi: 10.3390/jcm14041297 (PMC11856496; doi:10.3390/jcm14041297)
Supplement: Supplementary file 1 [file jcm-14-01297-s001.zip › jcm-3453100-supplementary.pdf]

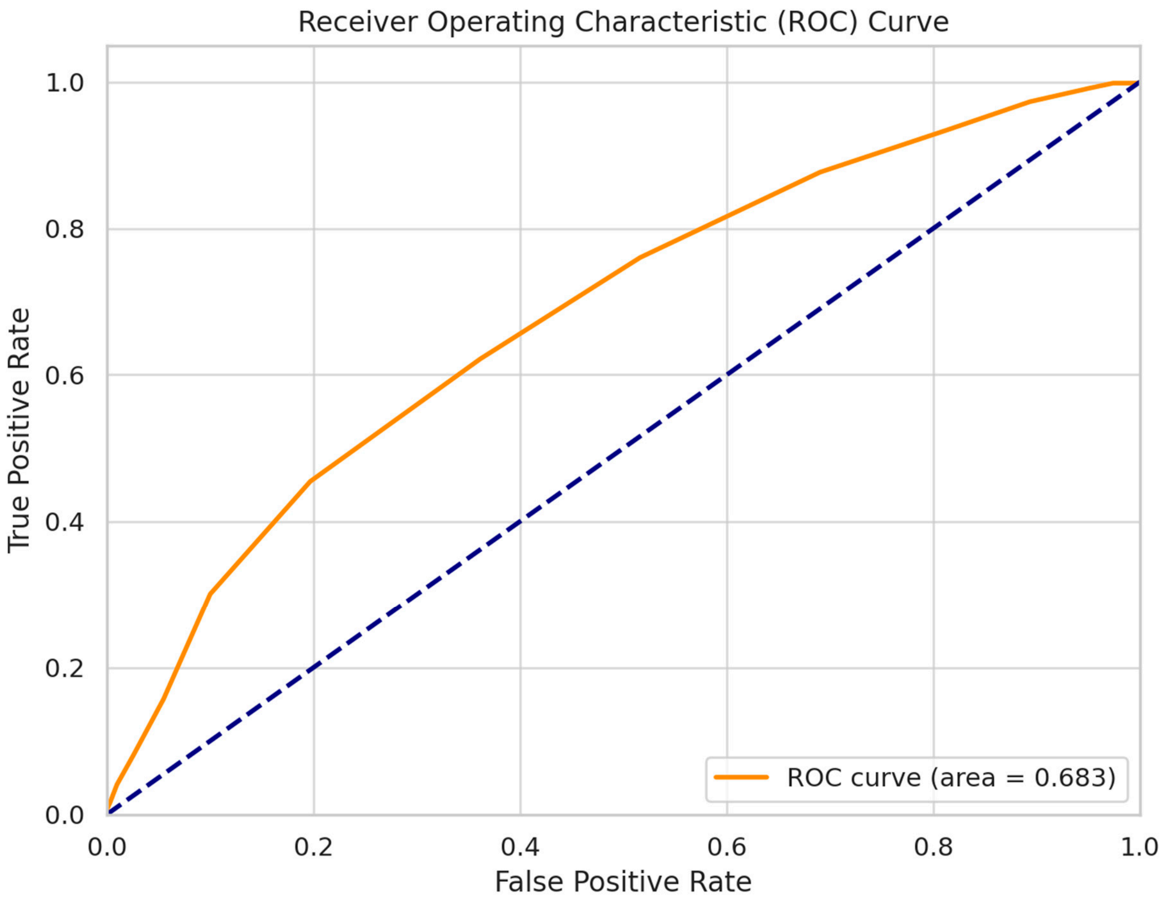

Supplementary Figure S1: Receiver Operating Characteristic (ROC) curve evaluating the performance of the risk score in predicting CAD in our cohort.

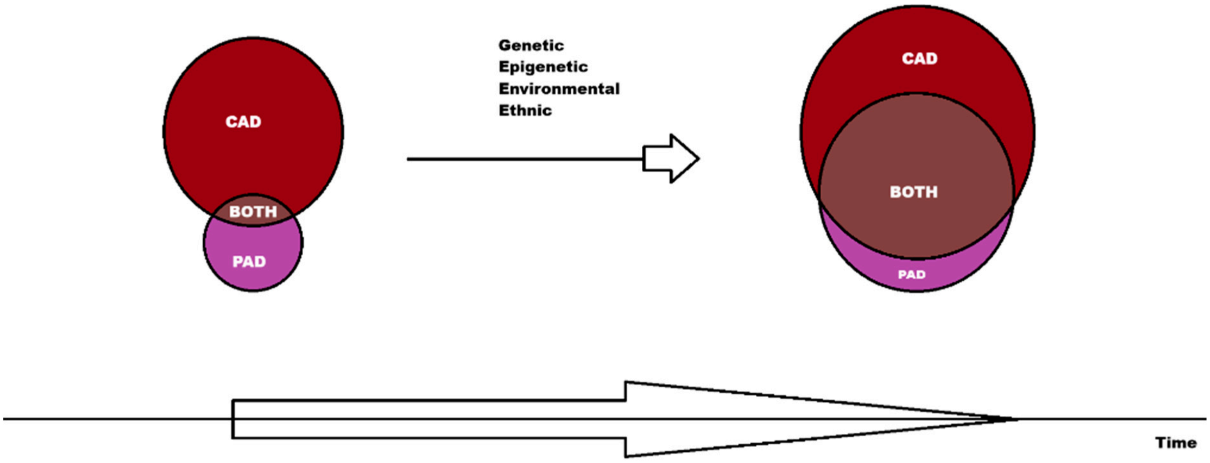

Supplementary Figure S2. This figure illustrates the temporal progression and relationship between coronary artery disease (CAD) and peripheral artery disease (PAD), highlighting the factors influencing their development and progression.

Supplementary Table S1. Variables used in the BG scoring system and other coronary artery disease (CAD) risk scoring models.

Scoring System	Variables
BG Study	ABI, carotid velocities, hypertension, smoking, diabetes, CKD, PAD, age
Diamond-Forrester Score [48]	Age, gender, type of chest pain (typical/atypical/non-anginal)
CAD Consortium Score [2]	Age, gender, type of chest pain, smoking, diabetes, and other clinical factors
Duke Clinical Score [49]	Symptoms, risk factors (smoking, diabetes), and stress test results
Framingham Risk Score [1]	Age, cholesterol (total and HDL), systolic blood pressure, smoking, and diabetes
CAD-RADS [51]	Coronary stenosis severity and plaque characteristics based on CCTA

Abbreviations: ABI, ankle-brachial index; CAD, coronary artery disease; CKD, chronic kidney disease; HDL, high-density lipoprotein; CCTA, coronary computed tomography angiography; PAD, peripheral arterial disease. CAD-RADS™ Coronary Artery Disease - Reporting and Data System.

Supplementary Table S2. Performance metrics of the BG scoring system compared to other coronary artery disease (CAD) risk scoring models.

Scoring System	AUC	Sensitivity	Specificity
BG-Score	0.683	97%	11%
Diamond-Forrester Score	~0.58	Moderate	Low
CAD Consortium Score	~0.75-0.78	~80-90%	~55-70%
Duke Clinical Score	~0.66	Moderate	Moderate
Framingham Risk Score	0.63-0.83	Variable	Variable
CAD-RADS	~0.86	High	Moderate

Abbreviations: AUC, area under the receiver operating characteristic curve; CAD, coronary artery disease, CAD-RADS™ Coronary Artery Disease - Reporting and Data System. Metrics include sensitivity (ability to identify CAD-positive patients) and specificity (ability to exclude CAD-negative patients.
